# Regional Inequality and Associated Factors of Emergency Medicine Beds Distribution in China

**DOI:** 10.3389/ijph.2024.1606812

**Published:** 2024-04-08

**Authors:** Change Xiong, Ying Xia, Huihui Chen, Jing Cheng

**Affiliations:** ^1^ School of Public Health, Wuhan University of Science and Technology, Wuhan, Hubei, China; ^2^ Institute of Nursing Research, Hubei Province Key Laboratory of Occupational Hazard Identification and Control, School of Medicine, Wuhan University of Science and Technology, Wuhan, Hubei, China

**Keywords:** regional inequality, emergency medicine beds, resources distribution, associated factors, Gini coefficient

## Abstract

**Objective:** The regional inequality of emergency medicine beds distribution has a great impact on population health as well as the accessibility of emergency services. This study aimed to explore the regional inequality of emergency medicine bed distribution and its influencing factors.

**Methods:** The Gini coefficient and health resource agglomeration were used to analyze the regional inequality of emergency medicine beds distribution by area from 2012 to 2021 in China. Grey correlation models were used to explore the factors influencing the regional inequality of emergency medicine beds distribution.

**Results:** From 2012 to 2021, Gini coefficients of emergency medicine beds distribution by geographic in China showed a worsening trend, rising from 0.6229 to 0.6636. The average HRAD index was 3.43 in the east and 0.44 in the west. Population structure factors have the greatest influence on the regional inequality of emergency medicine beds distribution.

**Conclusion:** Health resources allocation strategy only according to population size should be changed. In formulating policies for emergency medicine beds allocation should take into account population structure, financial structure of expenditure, the inequality of geographical distribution and so on.

## Introduction

Ensuring that residents enjoy equal medical services is an important goal for the high-quality development of China’s health system. The emergency medical system is an important high-quality medical resource throughout the world. It generally consists of pre-hospital emergency care, hospital emergency departments and intensive care units in China. Pre-hospital emergency services rely on emergency centers, however, emergency centers are very small amount in a region in China, and people mainly rely on hospital emergency departments for most of their urgent medical needs. As a multifunctional unit, emergency department is not only the core of the emergency medical system, but also plays an important role in the medical system. It shoulders the burden of dealing with acute and critical care, public health emergencies, mass trauma events and natural disasters. The global response to the new crown epidemic has shown that [1] effective and adequate emergency medical resources are essential to minimize the negative impact of an outbreak. A study showed that [2] timely and effective emergency medical services can reduce adult unintentional injury mortality by 80%, pediatric unintentional injury mortality by 60%, and mortality in low- and middle-income countries by 45%. Because China’s hierarchical medical system has not yet been fully established, patients who are unable to receive timely medical care in outpatient clinics choose to visit the emergency department (ED) as a medical-seeking strategy to satisfy their medical needs. The limited medical resources in the ED have led to serious overcrowding problems all the time [3]. There is an empirical evidence that [4] a better bed supply strategy can reduce overcrowding in the emergency department and improve the quality of emergency care.

The implementation of the “Healthy China” strategy had substantially increased the effective supply of medical resources in China. The number of emergency department beds nationwide increased from 25,038 to 52,668 in the past decade. Nevertheless, most of the existing strategies on the allocation of health resources are formulated on the basis of population in China, with less consideration given to geographical distribution. As a result, the distribution of resources shows more equal in terms of population, while inequality still exists among regions. Medical institutions, medical staff and beds, and other medical resources were distributed unequally by geographical with Gini coefficients exceeding 0.7 [5]. This leads to a large number of people living within the county having not enough resources for emergency care and poor access to healthcare [6]. The distribution of emergency care resources also shows urban-rural inequalities, with emergency care resources being particularly scarce in rural areas [7].

Factors affecting inequality in the allocation of healthcare resources are manifold [8, 9] including the system, the market, the economy, health insurance and so on. Some studies have shown that [10] factors such as the quantity of the children and elderly population, government investment in healthcare, the quantity of highly qualified population, healthcare expenditures, and government revenues have a large direct impact on the geographical distribution of health resources. Li et al [11, 12] results also found that economic development, urbanization, population aging, level of financial health expenditure and population size are key factors influencing the geographic distribution of medical resources in China. However, previous studies have not explored in depth the factors influencing the regional inequality of emergency medical resources, and the distribution of emergency medicine beds has a more prominent impact on the effectiveness and quality of emergency department care [7]. Moreover, the rationality of geographic distribution and spatial allocation of emergency care resources play a key role in guaranteeing the spatial accessibility of emergency care resources and reducing the morbidity and mortality rate of the population.

This study analyzes the regional inequity of the distribution of emergency medicine beds in 31 provinces as well as in the eastern, central, and western China from 2012 to 2021, and applies the grey correlation model to assess the influencing factors of regional inequity in the distribution of beds in emergency department. The results of the study help to provide actual evidence for adjusting emergency medicine beds allocation policy to optimize the geographic distribution of emergency medicine beds, which contributes to a balanced layout of high-quality medical resources in China.

## Methods

### Data Collection

The data were obtained from the 2013–2022 China Statistical Yearbook and China Health Statistical Yearbook, covering 31 provinces, autonomous regions and municipalities directly under the central government in China in this study.

### Defining Variables

In this study, we calculated the Gini coefficient of emergency medicine beds by area (per 10,000 km^2^) and the degree of agglomeration of geographic areas to represent the regional inequity of emergency medicine beds distribution, and explored the factors affecting the regional inequality of emergency medicine beds distribution from the economy, population and medical resources/indicators dimensions. The other control variables were selected and defined as shown in Table 1.

**TABLE 1:** Influencing factors of geographic equality of emergency beds. (China, 2012–2021).

Dimension	Variable	Explanation	Definition
Economy	*per capita* GDP	Gross regional Product/total population	X1
	Per capita disposable income	The income that residents may use for their free disposal includes income from wages and net income from business operations	X2
Population	Number of the children	Population aged 0–14	X3
	Number of the old	Population aged 65 and above	X4
	Proportion of urban population	Urban population/total population	X5
Medical resources/indicators	Government health expenditure as a proportion of total health expenditure	Government health expenditure/total health expenditure	X6
	Composition of practicing (assistant) physicians	The proportion of the number of practicing (assistant) physicians in the department of Emergency medicine to the number of total practicing (assistant) physicians in the department of hospital	X7
	Number of discharged patients	Number of people discharged after all hospitalizations in the Emergency Medicine Department during the reporting period	X8
	Outpatient and emergency visits	Number of outpatient visits in emergency medicine	X9

### Data Analysis

#### Gini Coefficient

In this study, the Gini coefficient (G) was applied to assess the inequality of the geographic distribution of emergency medicine beds. The closer the value of G is to 1, the more unequal it is. The formula used to calculate G is as follows:

$$G=\sum_{i=1}^{n}W_{i}Y_{i}+2\sum_{i=1}^{n}\left(W_{i}-W_{i}V_{i}\right)-1$$

where *Wi* is the proportion of each province’s geographic area to the total area of the country, *Yi* is the proportion of emergency medicine beds in each province to the total number of emergency medicine beds in the country, and *Vi* denotes the cumulative proportion of each indicator in each of the 31 provinces (ranked from smallest to largest in terms of the number of emergency medicine beds per 10,000 square kilometers). n represents the 31 provinces. These data were analyzed using Stata 26.0 and EXCEL 2016.

#### Health Resource Agglomeration Degree

Health resource agglomeration degree (HRAD) is a new indicator for evaluating the inequality of health resource distribution [13]. This study analyzed the clustering of emergency medicine beds in eastern, central, and western China. The eastern, central, and western regions are based on geographic location and level of economic development. The eastern region includes Hebei, Liaoning, Jiangsu, Zhejiang, Fujian, Shandong, Guangdong, Hainan, Beijing, Tianjin, and Shanghai; the central region includes Shanxi, Jilin, Heilongjiang, Anhui, Jiangxi, Henan, Hubei, and Hunan; and the western region includes Sichuan, Guizhou, Yunnan, Shaanxi, Gansu, Qinghai, Inner Mongolia, Guangxi, Tibet, Ningxia, and Xinjiang and Chongqing [14]. The formula for calculating the degree of agglomeration based on geographic area is:

$$HRAD_{i}=\frac{\left(HR_{i}/HR_{n}\right)\times 100\%}{\left(A_{i}/A_{n}\right)\times 100\%}=\frac{HR_{i}/A_{i}}{HR_{n}/A_{n}}$$

HRAD_i_ denotes the concentration of emergency medicine beds in a region i, HR_i_ denotes the number of emergency medicine beds, and A_i_ denotes the land area; An is the land area of the region at the upper level, and HR_n_ is the number of emergency medicine beds in the region at the upper level.

#### Grey Relational Analysis

Grey Relational Analysis (GRA) modeling, is a popular technique for decision analysis under multiple criteria decision making and uncertainty. Deng GRA, absolute GRA, and SS GRA are the three components of the GRA model. In this study, grey correlation analysis model is adopted to explore the impact of each index on emergency medicine bed inequality by region. Gini coefficient of emergency medicine bed by area (per 10,000 km^2^) from 2012 to 2021 is used to represent its regional inequality. Grey relational gradient (GRG) represents the outcomes of grey relational models. The absolute GRG and SSGRG models have values ranging from zero to one, whereas, Deng GRG has values ranging from 0.5 to 1. It is also considered highly associated if value is near to 1.

The algorithm for computing the Deng GRA is shown below [15].

$$\begin{array}{c} \gamma \left(X_{o},X_{i}\right)=\frac{1}{n}\sum_{k=1}^{n}\gamma \left(x_{o}\left(k\right),x_{i}\left(k\right)\right),\\ \gamma \left(x_{o}\left(k\right),x_{i}\left(k\right)\right)=\frac{\min_{i}\min_{k}\left|x_{o}\left(k\right)-x_{i}\left(k\right)\right|+\xi \max_{i}\max_{k}\left|x_{o}\left(k\right)-x_{i}\left(k\right)\right|}{\left|x_{o}\left(k\right)-x_{i}\left(k\right)\right|+\xi \max_{i}\max_{k}\left|x_{o}\left(k\right)-x_{i}\left(k\right)\right|} \end{array}$$

If X_0_ = (x_0_ (1), x_0_ (2), …, x_0_ (n)) is the underlying/reference sequence representing the dependent variable and Xi = (x_i_ (1), x_i_ (2), …, x_i_ (n)) is a set of comparison sequences representing the independent variables, a series of operations results in the grey correlation, γ_0i_ or γ (X_0_, X_i_), where ξ ∈ (0, 1) is the discriminant coefficient, the value of which is usually taken as 0.5. k is the sequence number of the data, and i is the number of influencing factors.

The algorithm for computing the absolute GRA is shown below [16].

$$\in_{oi}=\frac{1+\left|r_{o}\right|+\left|r_{i}\right|}{1+\left|r_{o}\right|+\left|r_{i}\right|+\left|r_{o}-r\right|}$$

$$r_{o}=\int_{1}^{n}X_{o}^{0}dt,r_{i}=\int_{1}^{n}X_{i}^{0}dt,r_{o}-r_{i}=\int_{1}^{n}\left(X_{o}^{0}-X_{i}^{0}\right)dt$$

The Second synthetic GRA (SSGRA) model can be produced by incorporating the given equation.

$$SSGRA=\theta Deng^{\prime }sGRA+\left(1-\theta \right)AbsoluteGRA$$

Whenever a decision-maker seeks an inclusive evaluation that combines the pros of both “Deng’s GRA” and “Absolute GRA” without endorsing one over another and so sets θ at 0.5, we assumed θ at 0.5 for our analysis [17].

## Results

### Regional Inequality in Emergency Medicine Beds Distribution

The results of the Gini coefficient by area allocation indicated that the overall geographic distribution of emergency beds was in a state of severe inequality from 2012 to 2021, with Gini coefficients above 0.6. The overall trend was increasing, from 0.6229 in 2012 to 0.6636 in 2021 (see Table 2).

**TABLE 2:** Gini coefficients for emergency beds distribution by area. (China, 2012–2021).

Year	Gini coefficient
2012	0.6229
2013	0.6275
2014	0.6312
2015	0.6376
2016	0.6399
2017	0.6383
2018	0.6466
2019	0.6460
2020	0.6533
2021	0.6636

**FIGURE 1:**
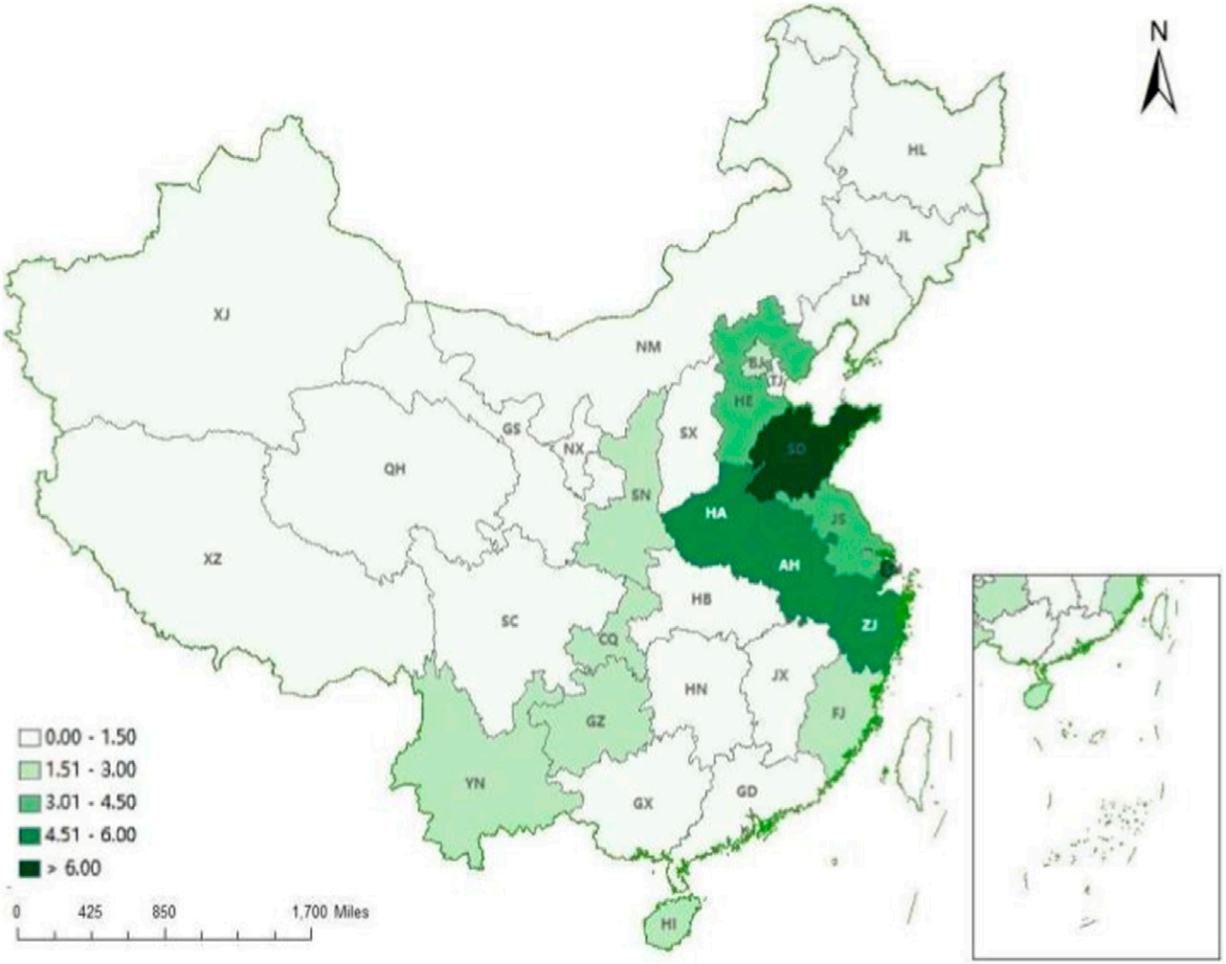
Distribution of regional aggregation of emergency beds in 31 provinces. (China, 2012).

**FIGURE 2:**
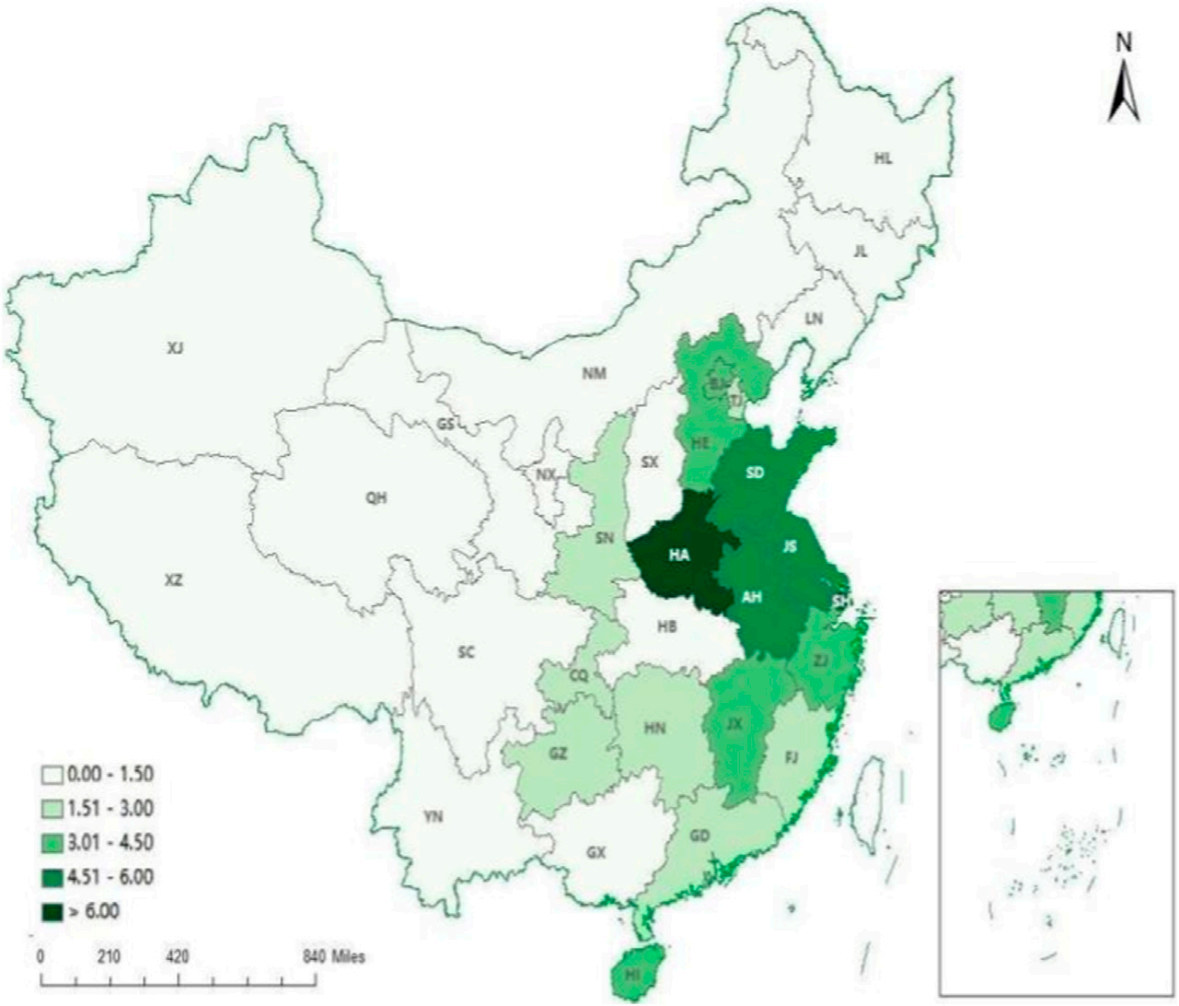
Distribution of regional aggregation of emergency beds in 31 provinces. (China, 2021). Note: BJ, Beijing; TJ, Tianjing; HE, Hebei; SX, Shanxi; NM, Inner Mongoria; SH, Shanghai; JS, Jiangsu; SD, Shandong; ZJ, Zhejiang; AH, Anhui; JX, Jiangxi; FJ, Fujian; GD, Guangdong; GX, Guangxi; HI, Hainan; HA, Henan; HB, Hubei; HN, Hunan; SN, Shaanxi; XJ, Xinjiang; NX, Ningxia; GS, Gansu; QH, Qinghai; CQ, Chongqing; SC, Sichuan; GZ, Guizhou; YN, Yunnan; XZ, Tibet; HL, Heilongjiang; JL, Jilin; LN, Liaoning.

From 2012 to 2021, the geographic concentration of emergency medicine beds less than 1 only was in the western China all over the country. The average HRAD index was 3.43 in the east and 0.44 in the west. The geographic clustering of emergency medicine beds in the eastern and central regions is greater than 1, indicating that emergency medicine beds in the eastern and central regions are more equally distributed compared to the western region. Overall, the geographic clustering degree in the eastern and western regions showed a decreasing trend, and the geographic clustering degree in the central region showed an increasing trend (see Table 3). As can be seen from Figures 1, 2, the geographic agglomeration of emergency medicine beds in the eastern region is the largest, with Shandong province and Shanghai city being more prominent. From 2012 to 2021, the emergency medicine beds resources have a tendency to cluster in the central region.

**TABLE 3:** Changes in geographic clustering of emergency beds. (China, 2012–2021).

Year	East	Central	West
2012	3.4860	1.4807	0.4931
2013	3.4977	1.5165	0.4824
2014	3.4851	1.6140	0.4604
2015	3.4399	1.7008	0.4462
2016	3.4000	1.7699	0.4354
2017	3.3639	1.7992	0.4339
2018	3.4542	1.8046	0.4185
2019	3.4420	1.8342	0.4131
2020	3.4025	1.9106	0.4005
2021	3.3523	1.9966	0.3872

### Analysis of Factors Influencing Regional Inequality in Emergency Beds

The results are shown in Table 4. According to the results of Deng’s GRA, the correlation degree of all factors was above 0.5, indicating that all factors were closely related to the geographical inequality of emergency beds. The results of SS GRA were consistent with those of Deng’s GRA. The top three factors were the number of 0–14 children, the composition of practicing (assistant) physicians and the proportion of urban population. The number of outpatient and emergency visits and the *per capita* disposable income of residents ranked eighth and ninth, respectively. According to the results of absolute correlation, the number of 0–14 children was found to have the strongest effect on regional equality of emergency medicine beds by attaining a grey score of 0.9821. The number of discharged patients and the number of outpatient and emergency patients had the least influence on the regional equality of emergency medicine beds with a grey score of 0.8861 and 0.8693, respectively.

**TABLE 4:** Grey correlation evaluation results. (China, 2012–2021).

Geographic equity of emergency medicine beds	Deng GRG	Rank	Absolute GRG	Rank	SSGRG	Rank
X4	0.6459	5	0.9400	5	0.7930	5
X1	0.5886	7	0.9165	6	0.7525	7
X5	0.8146	3	0.9763	2	0.8954	3
X3	0.9137	1	0.9821	1	0.9479	1
X2	0.5274	9	0.8891	7	0.7083	9
X6	0.7939	4	0.9644	4	0.8791	4
X8	0.6198	6	0.8861	8	0.7529	6
X7	0.8545	2	0.9724	3	0.9135	2
X9	0.5526	8	0.8693	9	0.7110	8

Note: X1, *per capita* GDP; X2, per capita disposable income; X3, number of the children; X4, number of the old; X5, proportion of urban population; X6, government health expenditure as a proportion of total health expenditure; X7, Composition of practicing (assistant) physicians; X8, number of discharged patients; X9, outpatient and emergency visits.

## Discussion

This study depicts provincial inequalities in the distribution of emergency medicine beds by geographic area, assesses and compares the extent to which population, economic, and medical resource factors are associated with regional inequality in emergency medicine beds, and provides a reference for regional allocation of emergency medicine beds. The results of our analysis report the following four key findings.

### Regional Inequities in Emergency Medicine Beds Show a Gradual Increasing Trend, 2012–2021

There are obvious regional differences in the overall distribution of emergency medicine beds from 2012 to 2021. Emergency medicine beds are mainly concentrated in the economically developed eastern region, while the distribution of emergency medicine beds in the relatively underdeveloped western region is obviously insufficient. The result is consistent with the results of the overall allocation of health resources [18]. Moreover, the inequality in the geographic distribution of emergency medicine beds has shown a trend of gradual deterioration. This may be attributed to China’s health resource allocation policy. For a long time, the Chinese government allocated health resources mainly according to the living population. The result of this health policy is that equality in health resources is better distributed by population than by geographic area [19]. However, this allocation strategy neglects the special characteristics of remote areas. The number of people living in remote areas is small, the allocation of medical resources according to population is correspondingly small. This leads to the scattered residents will not be able to obtain timely emergency services due to the large geographic area of the province.

Distribution of emergency medicine beds according to geographic shows poor equality and needs more attention. Especially for sparsely populated provinces with large geographic areas, it is an important strategy allocating resources by area to promote the accessibility and equality of health services for the population. Some countries have adopted the allocation of medical resources according to area. For example, the government of Japan [20] had set up a three-tier healthcare medical circle based on geographical characteristics and demand for health services, with strict control over the number of health institutions and hospital beds. In Singapore [21], all public medical institutions are divided into two medical groups, one in the east and the other in the west, according to their geographical location and size. The allocation of emergency medicine beds needs to take into account geographic size, not just the residential population, and the trend of worsening geographic inequality is likely to continue if the allocation strategy is not changed.

### Population Structure Factors Are the Most Significant Factors Influencing Regional Inequality in the Distribution of Emergency Medicine Beds

The grey correlations showed that population structure factors has a greatest impact on the regional inequality of emergency medicine department beds, which is consistent with the findings of Wu et al [5]. Children and the elderly population, who are physically weaker due to age, tend to be at higher risk of acute illness and injury, and have a greater need for emergency care. The health problem of left-behind children in rural or remote areas deserves attention, and as a vulnerable group in terms of health, they may need more emergency medical resources to meet their complex medical needs [22]. Some findings [23] have demonstrated that the more adequate emergency medical resources such as beds, the lower the risk of adverse outcomes for elderly patients.

There is a correlation between the proportion of urban population and the regional inequality of emergency medicine beds. Since the reform and opening up, the acceleration of urbanization has created difficulties in the reasonable distribution of medical resources [24]. The gap is widening between urban and rural areas in terms of the supply of health resources and the demand for health services. High-quality emergency medical resources are mainly concentrated in large hospitals in big cities, which may be related to the “siphoning effect” on emergency medical resources as to the high population density of urban areas [22]. Overall, multiple population factors can influence the regional inequality of emergency medicine beds.

### Increasing Government Health Expenditure as a Proportion of Total Health Costs Is Beneficial to Reducing Regional Inequalities in the Distribution of Emergency Medicine Beds

The results indicated expanding government’s health spending’s share of total health costs rather than only increasing government’s health spending in order to have a mitigating effect on regional inequality in acute care bed. This is in consistent with the findings of Cashin et al [25]. The government’s ability to utilize its finances has a significant impact on the distribution of health resources, and it is necessary to attach great importance to building financial capacity in the allocation of health resources. Poor areas are often less likely to have adequate access to emergency care than wealthier areas. There is a significant gap in the equality of medical resource distribution among provinces. The scarcity and uneven distribution of emergency care resources is also a serious problem in low- and middle-income countries [26]. Increasing the proportion of government health expenditures can strengthen the construction of medical facilities in remote and underdeveloped provinces and reduce the gap in the distribution of emergency medicine beds among provinces.

In a word, local governments should optimize the structure of financial expenditure, increase the proportion of government health expenditure in the total cost of health, expand the coverage of emergency medical resources, and decrease the inequality in the regional distribution of emergency medicine beds.

### The Composition of Practicing (Assistant) Physicians Is Closely Related to the Regional Inequality of Emergency Medicine Beds Distribution

The findings showed that the composition of practicing (assistant) physicians in the emergency medicine department was strongly associated with the inequality of the geographic distribution of beds. The study of Horev T et al supported our findings [27]. Licensed (assistant) physicians play a more prominent role as an integral part of the acute care delivery system [28]. The distribution of practicing (assistant) physicians in the emergency department will have an impact on the effectiveness, accessibility and sustainability of emergency medical services. However, the western region lags behind in terms of environmental conditions, economic level and compensation compared with the central and eastern regions, the trend of physician mobility is more urban-centered, which will exacerbate the geographic inequality in the allocation of beds [5]. The government needs to make targeted plans and strategies in undeveloped provinces to reduce geographic inequalities in the distribution of emergency department physicians and beds and to narrow the gap in the level of emergency medical services among regions.

### Conclusion

Gradual deterioration in the inequality of regional distribution of emergency medicine beds in China from 2012 to 2021. Three factors, population structure, economy level and medical resources/indicators had different influence on the disparity of emergency medicine beds distribution, and the population structure having the greatest degree of association with the regional inequality of emergency medicine beds. The government should consider the inequality of geographical distribution when formulating resource allocation policies and optimize financial structure expenditures. In undeveloped areas, it is important to take practical and feasible simulation measures to increase the number of emergency physicians. In regions with a high proportion of vulnerable populations such as the elderly and children, or a high proportion of the urban population, the number of emergency medicine beds should be increased.

### Enlightenment

The results of this study provide a basis for promoting the geographic equality, service accessibility and sustainable development of emergency medical resources in developing countries. We call on countries around the world to pay great attention to the spatial inequality of emergency medical resources to improve the emergency medical service system accessibility. This study suggests that both population equity and spatial accessibility equity should be considered simultaneously in the practice of allocation of emergency medicine beds. Moreover, in areas where spatial distribution of emergency medicine beds is unequitable but the distribution is equitable according to population, priority should be given to reducing spatial distribution unfairness in emergency medical resource planning. Conversely, priority should be given to reducing population distribution unfairness.

### Limitation and Future Research

This is an exploratory study only explored the association between macro factors such as population, economy, and medical factors and regional inequality of emergency medicine beds distribution. In the future, attention could be paid to the interaction between other factors such as individual aspects and macro influences. In addition, Due to the availability of data, this study did not test the results robust of grey correlation analysis. Future studies should consider more factors and use more complete database to comprehensively evaluate the distribution of emergency medicine beds.

## Data Availability

The survey data collected and analyzed during the current study are available from the corresponding author on reasonable request.

## References

[1] Wang X Wu W Song P He J. An International Comparison Analysis of Reserve and Supply System for Emergency Medical Supplies Between China, the United States, Australia, and Canada. Biosci Trends (2020) 14:231–40. 10.5582/bst.2020.03093 32389940

[2] Wong HT Lin TK Lin JJ. Identifying Rural-Urban Differences in the Predictors of Emergency Ambulance Service Demand and Misuse. J Formos Med Assoc (2019) 118:324–31. 10.1016/j.jfma.2018.05.013 29908869

[3] Tsai JCH Weng SJ Liu SC Tsai YT Gotcher DF Chen CH Adjusting Daily Inpatient Bed Allocation to Smooth Emergency Department Occupancy Variation. Healthcare (Basel) (2020) 8:78. 10.3390/healthcare8020078 32231146 PMC7349152

[4] Khare RK Powell ES Reinhardt G Lucenti M. Adding More Beds to the Emergency Department or Reducing Admitted Patient Boarding Times: Which Has a More Significant Influence on Emergency Department Congestion? Ann Emerg Med (2009) 53:575–85. 10.1016/j.annemergmed.2008.07.009 18783852

[5] Wu X Zhang Y Guo X. Research on the Equity and Influencing Factors of Medical and Health Resources Allocation in the Context of COVID-19: A Case of Taiyuan, China. Healthcare (Basel) (2022) 10:1319. 10.3390/healthcare10071319 35885847 PMC9324996

[6] Neville S Napier S Adams J Shannon K. Accessing Rural Health Services: Results From a Qualitative Narrative Gerontological Study. Australas J Ageing (2020) 39:e55–e61. 10.1111/ajag.12694 31254326 PMC7079086

[7] Jin K Zhang H Seery S Fu YY Yu SS Zhang LL Comparing Public and Private Emergency Departments in China: Early Evidence From a National Healthcare Quality Survey. Int J Health Plan M (2020) 35:581–91. 10.1002/hpm.2968 31721297

[8] Shi BG Fu YT Bai XD Zhang XY Zheng J Wang YP Spatial Pattern and Spatial Heterogeneity of Chinese Elite Hospitals: A Country-Level Analysis. Front Public Health (2021) 9:710810. 10.3389/fpubh.2021.710810 34604156 PMC8481595

[9] Love-Koh J Griffin S Kataika E Revill P Sibandze S Walker S. Methods to Promote Equity in Health Resource Allocation in Low- and Middle-Income Countries: An Overview. J Glob Health (2020) 16:6. 10.1186/s12992-019-0537-z PMC6958737 31931823

[10] Zhou Y Zhao K Han J Zhao S Cao J. Geographical Pattern Evolution of Health Resources in China: Spatio-Temporal Dynamics and Spatial Mismatch. Trop Med Infect Dis (2022) 7:292. 10.3390/tropicalmed7100292 36288033 PMC9609797

[11] Guo QB Luo K Hu RD. The Spatial Correlations of Health Resource Agglomeration Capacities and Their Influencing Factors: Evidence From China. Int J Env Res Pub Health (2020) 17:8705. 10.3390/ijerph17228705 33238597 PMC7700579

[12] Li J Chen X Han X Zhang G. Spatiotemporal Matching Between Medical Resources and Population Ageing in China From 2008 to 2017. BMC Public Health (2020) 20:845. 10.1186/s12889-020-08976-z 32493251 PMC7268461

[13] Chen J Lin ZC Li L Wang YY Pan Y Yang J Ten Years of China’s New Healthcare Reform: A Longitudinal Study on Changes in Health Resources. BMC Public Health (2021) 21:2272–13. 10.1186/s12889-021-12248-9 34903184 PMC8670033

[14] Dai G Li R Ma S. Research on the Equity of Health Resource Allocation in TCM Hospitals in China Based on the Gini Coefficient and Agglomeration Degree: 2009-2018. Int J Equity Health (2022) 21:145. 10.1186/s12939-022-01749-7 36199086 PMC9534739

[15] Liu SF Zhang HY Yang YJ. Explanation of Terms of Grey Incidence Analysis Models. Grey Syst (2017) 7:136–42. 10.1108/gs-11-2016-0045

[16] Liu SF Xie NM Forrest J. Novel Models of Grey Relational Analysis Based on Visual Angle of Similarity and Nearness. Grey Sys (2011) 1:8–18. 10.1108/20439371111106696

[17] Rehman E Rehman S. Particulate Air Pollution and Metabolic Risk Factors: Which Are More Prone to Cardiac Mortality. Front Public Health (2022) 10:995987. 10.3389/fpubh.2022.995987 36339190 PMC9631442

[18] Li Z Yang L Tang S Bian Y. Equity and Efficiency of Health Resource Allocation of Chinese Medicine in Mainland China: 2013-2017. Front Public Health (2020) 8:579269. 10.3389/fpubh.2020.579269 33384979 PMC7769806

[19] Yao H Zhan C Sha X. Current Situation and Distribution Equality of Public Health Resource in China. Arch Public Health (2020) 78:86. 10.1186/s13690-020-00474-3 32983449 PMC7507592

[20] Ishikawa T Nakao Y Fujiwara K Suzuki T Tsuji S Ogasawara K. Forecasting Maldistribution of Human Resources for Healthcare and Patients in Japan: A Utilization-Based Approach. BMC Health Serv Res (2019) 19:653. 10.1186/s12913-019-4470-x 31500619 PMC6734478

[21] Lim MK. Shifting the Burden of Health Care Finance: A Case Study of Public-Private Partnership in Singapore. Health Policy (2004) 69:83–92. 10.1016/j.healthpol.2003.12.009 15484609

[22] Yang C Cui D Yin S liu X Yang Y Ke X Fiscal Autonomy of Subnational Governments and Equity in Healthcare Resource Allocation: Evidence From China. Front Public Health (2022) 10. 10.21203/rs.3.rs-1765344/v1 PMC9561467 36249207

[23] Ukkonen M Jamsen E Zeitlin R Pauniaho SL. Emergency Department Visits in Older Patients: A Population-Based Survey. BMC Emerg Med (2019) 19:20. 10.1186/s12873-019-0236-3 30813898 PMC6391758

[24] Du M Zhao Y Fang T Fan L Zhang M Huang H Evaluating the Inequality of Medical Resource Allocation Based on Spatial and Non-Spatial Accessibility: A Case Study of Wenzhou, China. Sustainability (2022) 14:8331. 10.3390/su14148331

[25] Cashin C Gatome-Munyua A. The Strategic Health Purchasing Progress Tracking Framework: A Practical Approach to Describing, Assessing, and Improving Strategic Purchasing for Universal Health Coverage. Health Syst Reform (2022) 8:e2051794. 10.1080/23288604.2022.2051794 35446186

[26] Ouma PO Maina J Thuranira PN Macharia PM Alegana VA English M Access to Emergency Hospital Care Provided by the Public Sector in Sub-Saharan Africa in 2015: A Geocoded Inventory and Spatial Analysis. Lancet Glob Health (2018) 6:e342–50. 10.1016/S2214-109X(17)30488-6 29396220 PMC5809715

[27] Horev T Pesis-Katz I Mukamel DB. Trends in Geographic Disparities in Allocation of Health Care Resources in the US. Health Policy (2004) 68:223–32. 10.1016/j.healthpol.2003.09.011 15063021

[28] Bai G Kelen GD Frick KD Anderson GF. Nurse Practitioners and Physician Assistants in Emergency Medical Services Who Billed Independently, 2012-2016. Am J Emerg Med (2019) 37:928–32. 10.1016/j.ajem.2019.01.052 30733103

